# Distribution of Glutathione-Stabilized Gold Nanoparticles in Feline Fibrosarcomas and Their Role as a Drug Delivery System for Doxorubicin—Preclinical Studies in a Murine Model

**DOI:** 10.3390/ijms19041021

**Published:** 2018-03-29

**Authors:** Katarzyna Zabielska-Koczywąs, Anna Wojtalewicz, Ewelina Użarowska, Agata Klejman, Agata Wojtkowska, Izabella Dolka, Marek Wojnicki, Katarzyna Sobczak, Michał Wójcik, Haifa Shen, Mauro Ferrari, Roman Lechowski

**Affiliations:** 1 Department of Small Animal Diseases with Clinic, Faculty of Veterinary Medicine, Warsaw University of Life Sciences, Nowoursynowska 159c, 02-787 Warsaw, Poland; aniawojt@hotmail.com (A.W.); agata_wet@interia.pl (A.W.); roman_lechowski@sggw.pl (R.L.); 2 Laboratory of Animal Models, Nencki Institute of Experimental Biology, Polish Academy of Sciences, Pasteura 3, 02-093 Warsaw, Poland; e.uzarowska@nencki.gov.pl (E.U.); agata.klejman@gmail.com (A.K.); 3 Postgraduated School of Molecular Medicine, Warsaw Genomics, Żwirki i Wigury 93, 02-089 Warsaw, Poland; 4 Department of Pathology and Veterinary Diagnostics, Faculty of Veterinary Medicine, Warsaw University of Life Sciences, Nowoursynowska 159, 02-787 Warsaw, Poland; izabella_dolka@sggw.pl; 5 Faculty of Non-Ferrous Metals, AGH University of Science and Technology, Mickiewicza 30, 30-059 Kraków, Poland; marekw@agh.edu.pl; 6 Faculty of Chemistry, University of Warsaw, Pasteura 1, 02-093 Warsaw, Poland; katarzynasobczak@student.uw.edu.pl (K.S.); mwojcik@chem.uw.edu.pl (M.W.); 7 Department of Nanomedicine, Houston Methodist Hospital Research Institute, 6670 Bertner Ave., Houston, TX 77030, USA; hshen@houstonmethodist.org (H.S.); mferrari@houstonmethodist.org (M.F.); 8 Department of Medicine, Weill Cornell Medicine, 1320 York Avenue, New York, NY 10065, USA

**Keywords:** cats, confocal microscopy, doxorubicin, fibrosarcoma, gold nanoparticles, ICP-MS, Ki-67, murine model, tumor-associated macrophages

## Abstract

Feline injection site sarcomas (FISS) are malignant skin tumors with high recurrence rates despite the primary treatment of radical surgical resections. Adjunctive radiotherapy or chemotherapy with doxorubicin is mostly ineffective. Cellular and molecular causes of multidrug resistance, specific physio-chemical properties of solid tumors impairing drug transport, and the tumor microenvironment have been indicated for causing standard chemotherapy failure. Gold nanoparticles are promising imaging tools, nanotherapeutics, and drug delivery systems (DDS) for chemotherapeutics, improving drug transport within solid tumors. This study was conducted to assess the distribution of 4-nm glutathione-stabilized gold nanoparticles in FISS and their influence on kidney and liver parameters in nude mice. The role of gold nanoparticles as a doxorubicin DDS in FISS was examined to determine the potential reasons for failure to translate results from in vitro to in vivo studies. Grade III tumors characterized by a large area of necrosis at their core displayed positive immuneexpression of tumor-associated macrophages (TAM) at both the periphery and within the tumor core near the area of necrosis. Gold nanoparticles did not cause necrosis at the injection site and had no negative effect on liver and kidney parameters in nude mice. Gold nanoparticles accumulated in the tumor core and at the periphery and co-internalized with TAM—an important observation and potential therapeutic target warranting further investigation. The large area of necrosis and high immunoexpression of TAM, indicating “pro-tumor macrophages”, may be responsible for FISS tumor progression and therapeutic failure. However, further studies are required to test this hypothesis.

## 1. Introduction

Feline injection site sarcomas (FISS) are malignant solid tumors with a high recurrence rate (ranging from 14–69%) despite performing radical surgery (with 3–5 cm of “clean” margin), which is the primary method of treatment. Adjunctive radiotherapy or chemotherapy is also mostly ineffective. Doxorubicin (Dox) is the drug of choice in soft tissue sarcomas, including FISS; nevertheless, its effectiveness varies between clinical trials [1]. Chemotherapy failure is often indicated as a reason for high activity and up-regulation of ATP-dependent transporters, especially P-glycoprotein (P-gp) or multidrug resistance proteins (MRP) [2]. Nevertheless, despite the cellular and molecular causes of multidrug resistance (MDR), the tumor microenvironment and specific physio-chemical properties of solid tumors that cause imperfect drug delivery and transport have been recently indicated as important causes of standard chemotherapy failure. For effective concentration of the therapeutic agent within target cells, three requirements should be met: transport within vessels, across walls of blood vessels, and through interstitial space within the tumor [3]. Tumor architecture, especially high collagen content, and the unperfused regions with low oxygen pressure and low pH that lead to necrotic tissue are major determinants of imperfect drug distribution. Limited drug access results in drug concentrations that are too low in hypoxic cells, where most anticancer agents are also less active [4,5]. Furthermore, this causes a vicious circle as hypoxia, which is an indicator of the malignant phenotype, is involved in up-regulating genes responsible for MDR, including *ABCB1* encoding P-gp.

Colloid gold nanoparticles (Au-NPs) of various shapes (nanospheres, nanorods, nanoshells, and nanocages) are promising imaging tools, nanotherapeutics (through selective targeting of cancerous cells and achieving cell damage by photodynamic therapy), and drug delivery systems (DDS) for anticancer agents [6,7,8,9,10,11]. It has been proven that Au-NPs that are smaller than 10 µm in size can penetrate the tumor core easier than larger Au-NPs; the tumor core is often impossible to reach with standard chemotherapeutic agents. Moreover, Au-NPs were shown to bypass P-gp and, when conjugated to Dox, enhance the cytotoxic effect of free Dox in feline fibrosarcoma cell lines with high P-gp activity [12]. The aim of the study presented here was to assess the distribution of 4 nm glutathione-stabilized gold nanoparticles (Au-GSH) in feline fibrosarcomas and their influence on selected kidney and liver parameters in nude mice. Moreover, the role of Au-GSH as a DDS for Dox in FISS was assessed, indicating the possible reasons for failure to translate results from in vitro to in vivo studies.

## 2. Results

**Figure 1:**
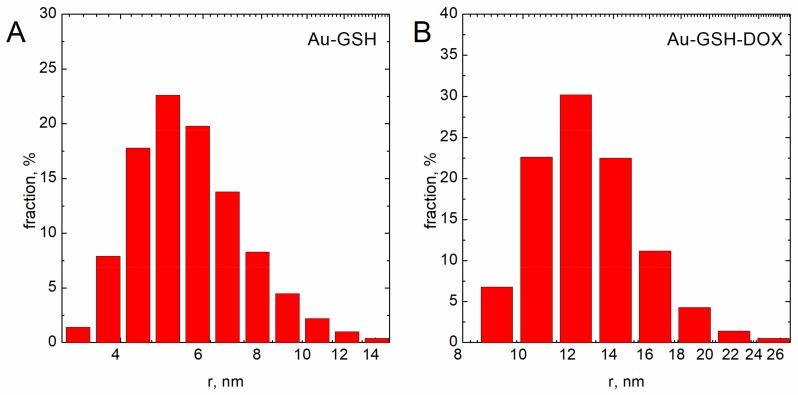
(**A**) Au-GSH size and size distribution. (**B**) Nanoparticle size and size distribution after linking doxorubicin.

**Figure 2:**
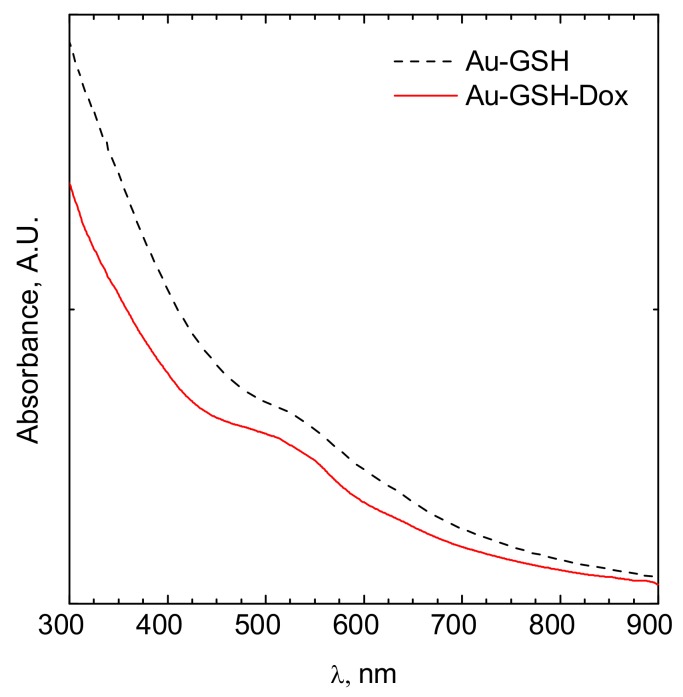
UV-Vis spectrum of a colloidal suspension of gold nanoparticles modified by glutathione (black dotted line) and doxorubicin (red solid line).

The hydrodynamic diameter of obtained nanoparticles after each step of synthesis was analyzed using the dynamic light scattering (DLS) method. To properly determine particle size, the density of the solution-obtained colloids was measured. The results obtained are shown in Figure 1. In the case of colloidal suspensions, the density of the system was identical to the density of the dispersant. The UV-Vis analysis of the obtained colloids after their modification is shown in Figure 2. The zeta potential determined using this equipment was equal to −52.9 ± 10.6 mV and −24.6 ± 11.9 mV for Au-GSH and Au-GSH-Dox, respectively.

**Figure 3:**
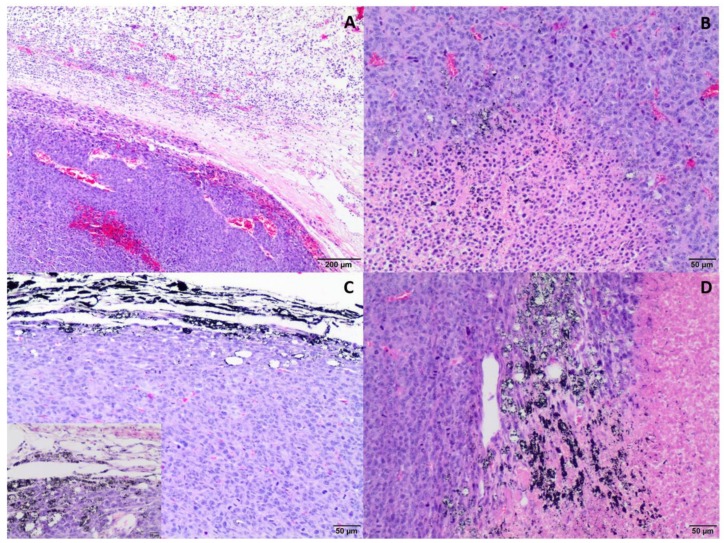
Representative images of feline injection site sarcoma (FISS) growth in immunodeficient mice. HE staining. Visible marked inflammatory infiltration in the periphery of the tumor (**A**) and the necrotic area in the tumor core (**B**). Au-GSH precipitate visible in Au-GSH-Dox-treated mice tumor periphery (**C**) and in the tumor core (**D**). Scale bar indicates 200 µm (**A**), 50 µm (**B**–**D**) and 20 µm (insert).

**Figure 4:**
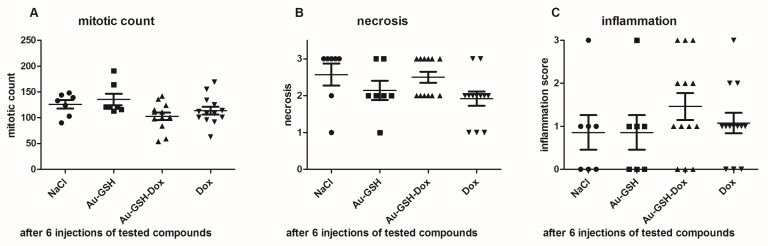
Statistical analyses of mitotic count (**A**), area of necrosis (**B**) and inflammation (**C**) in tumors after six injections of the tested compounds. Results are shown as the mean ± SD.

**Figure 5:**
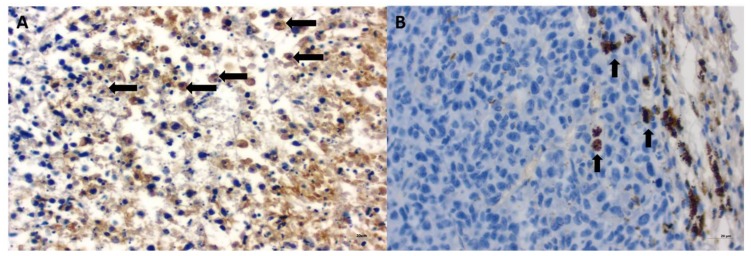
Immunoexpression of tumor-associated macrophages (TAM) in FISS growth in immunodeficient mice treated with Au-GSH-Dox in the tumor core close to the area of necrosis (**A**) and at the tumor periphery (**B**). Visible internalization of Au-GSH with TAM (**B**). Arrows indicate TAM. Bar graph 20 µm.

Tumor growth was obtained 5 days after neoplastic cell inoculation in 100% of animals (46 of 46 mice). HE staining confirmed typical hallmarks for grade III feline injection site sarcomas including an area of ovoid and spindle cells forming interwoven bundles, collagenous stroma, a large area of necrosis, and a high number of mitotic figures (Figure 3 and Figure 4A) (Table 1). In all tumors, an area of necrosis was visible in the tumor core, far from blood vessels, with no significant differences between treatment groups (Figure 4B). Scant to marked inflammatory infiltration was visible mostly at the peripheral part of the tumors (Figure 3A and Figure 4C). Moreover, tumors were characterized by high immunoexpression of TAM in the subcutaneous tissue surrounding the tumors (Figure 5B) and mostly close to the area of necrosis (Figure 5A).

**Figure 6:**
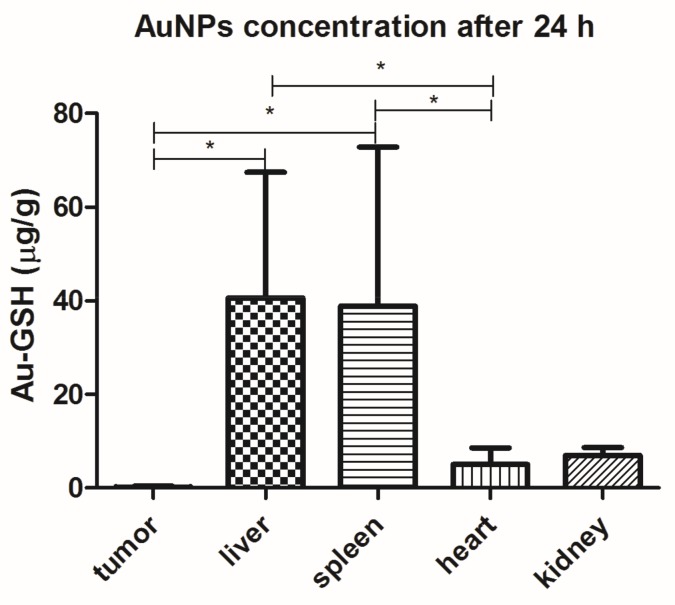
Au-GSH organ distribution (µg/g) after a single IV injection of Au-GSH in nude mice. Results are shown as the mean ± SD. (*n* = 6, *p* ≤ 0.05 marked with *).

Au-GSH accumulated mainly in the liver (40.6 ± 26.8% µg/g) and spleen (38.9 ± 33.9 µg/g), followed by the kidney and heart, after a single IV injection. Only 0.16 ± 0.24 µg/g of Au-GSH was found in the tumor tissue 24 h after IV injection of Au-GSH (Figure 6).

**Figure 7:**
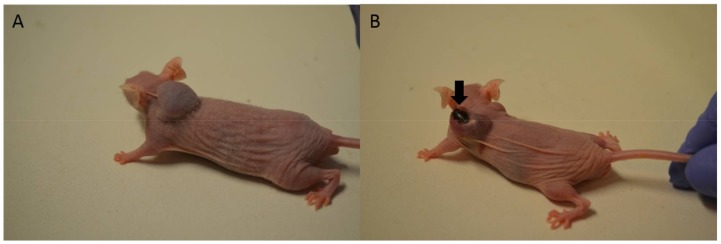
Macrophotographs of tumor growth on mice without (**A**) and with (**B**) visible skin necrosis in the place of intratumoral injections of Au-GSH-Dox (**A**) and Dox (**B**). Arrow indicates skin necrosis.

**Figure 8:**
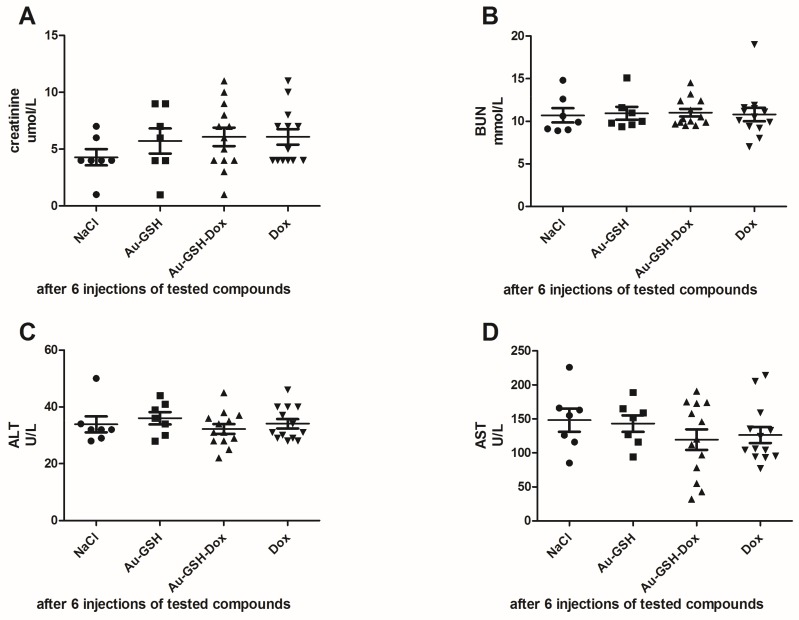
Statistical analyses of the levels of creatinine (**A**); BUN (**B**); ALT (**C**) and AST (**D**) in mice between treatment groups.

Skin necrosis was visible at the injection site in all mice that received free Dox, mostly after the fifth or sixth intratumoral injection (Figure 7B). In the case of Au-GSH-Dox-treated mice, most of the mice (ten of twelve) did not develop visible skin necrosis (Figure 7A) and only two developed skin necrosis at the injection site after the last (sixth) intratumoral injection of the test compound. Skin necrosis was not observed in any mouse from the control group nor mice from the Au-GSH-treated group. There were no statistically important differences in the levels of creatinine and BUN, nor AST and ALT between the treated groups after six intratumoral injections of the test compounds (Figure 8).

**Figure 9:**
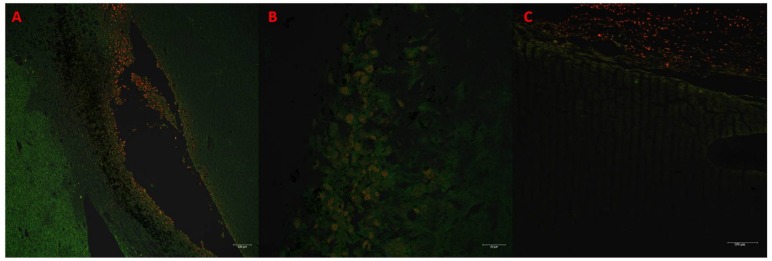
Confocal microscopy images presenting high Dox accumulation in the tumor core after treatment with Au-GSH-Dox (**A**,**B**). Bar graph 100 µm and 20 µm, respectively. Confocal microscopy image presenting Dox accumulation in the periphery of the tumor after treatment with Dox alone (**C**). Bar graph 100 µm.

**Figure 10:**
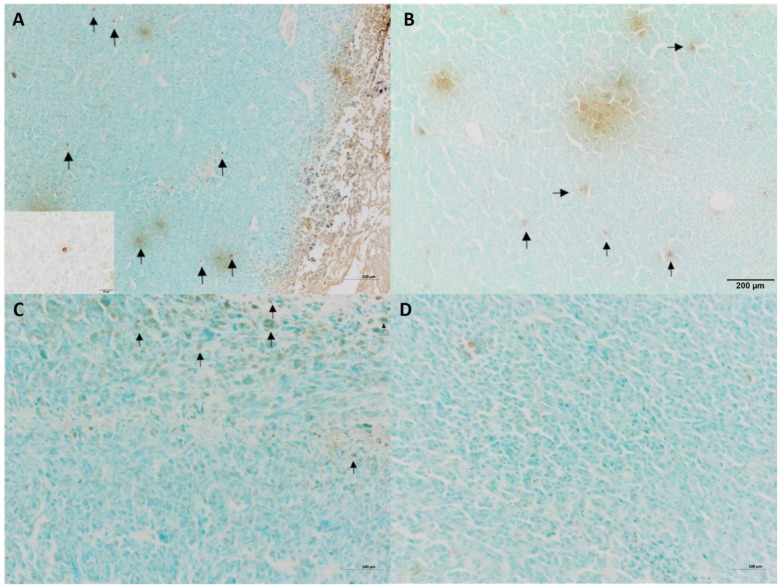
TUNEL staining of tumors treated with Au-GSH-Dox (**A**,**B**) and Dox alone (**C**,**D**) in tumor core and the periphery of tumors. Arrows indicate apoptotic bodies. Bar graph 100 µm (**A**,**C**,**D**) and 200 µm (**B**). Insert 20 µm.

**Figure 11:**
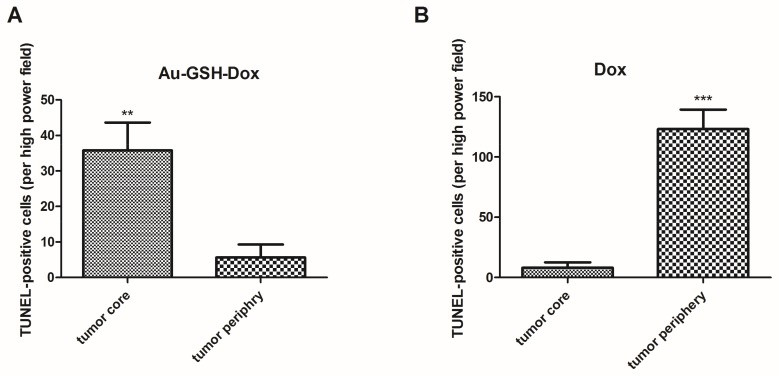
Statistical analysis of TUNEL-positive cells per HPF in the tumor core and periphery of tumors after six injections of Au-GSH-Dox (**A**) and Dox alone (**B**). Results are presented as the mean with SEM. *p* ≤ 0.01 is marked with **, while *p* ≤ 0.001 is marked with ***.

In tumors treated with Au-GSH-Dox or Au-GSH, precipitates of Au-NPs were visible within the tumor border in all tumors (Figure 3C) and in most cases in the core of tumors within the area of necrosis (Figure 3B) (Table 1). In the same regions, immunoexpression of TAM was observed (Figure 5A,B), as well as internalization of TAM and Au-GSH (Figure 5B). In tumors treated with Au-GSH-Dox, Dox was always visible close to Au-NP precipitates and was mainly localized in the tumor core (Figure 9A,B). Significantly (*p* ≤ 0.01) higher numbers of apoptotic bodies were visible in the tumor core in comparison to the tumor periphery in Au-GSH-Dox-treated mice compared to Dox-treated mice where apoptotic bodies were mostly visible in the tumor periphery (Figure 10 and Figure 11). Similar to the localization of apoptotic bodies in tumors treated with Dox alone, Dox was mostly visible at the periphery of tumors (Figure 9C).

**Figure 12:**
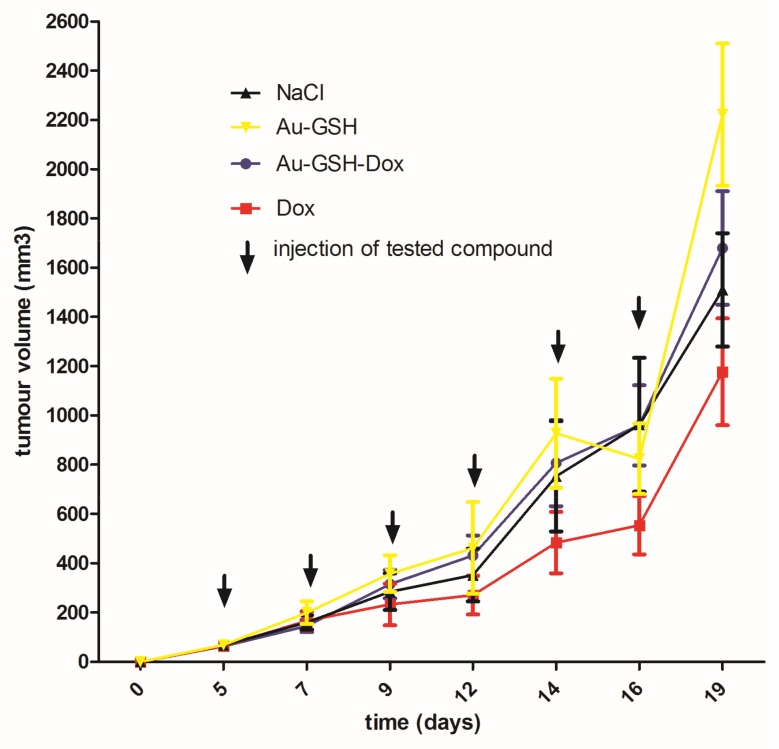
Tumor growth curve following treatment with tested compounds. Data are presented as the mean with SD.

**Figure 13:**
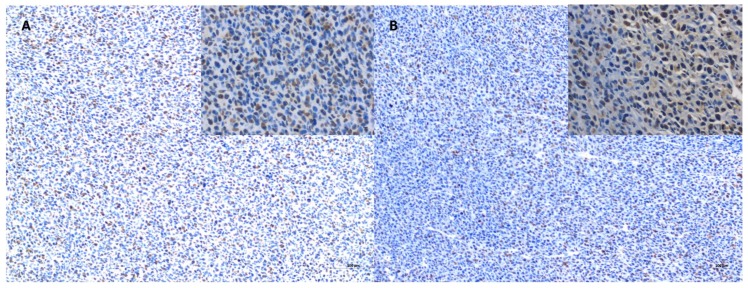
Immunoexpression of Ki-67 in the tumor core after treatment with Au-GSH-Dox (**A**) and Dox (**B**). Bar graph 100 µm and 20 µm (inserts).

**Figure 14:**
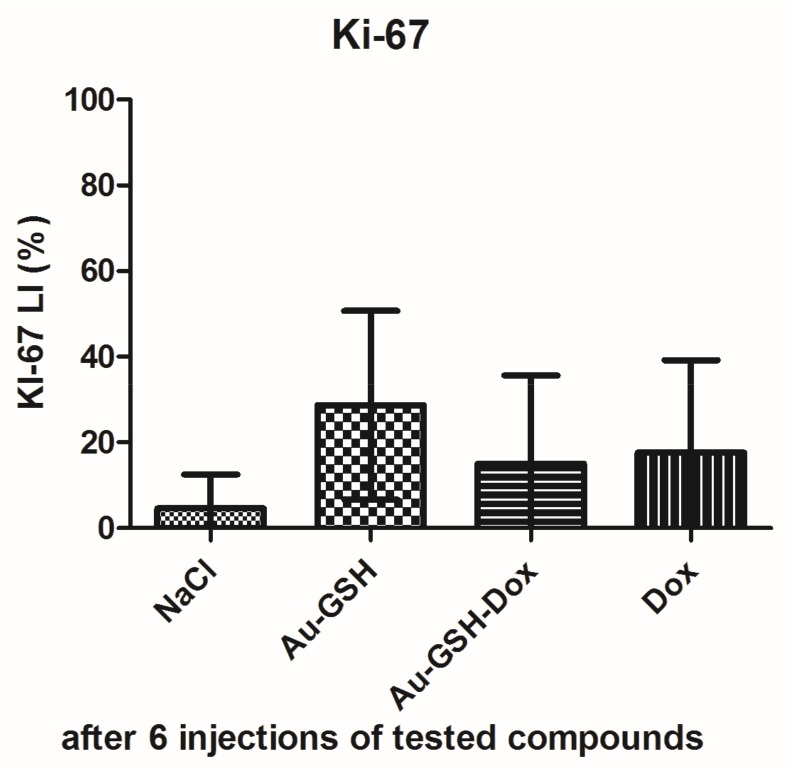
Statistical analysis of Ki-67 LI in FISS after six intratumoral injections of test compounds. Data are presented as the mean with SD.

Tumor progression was visible after treatment with test compounds within all groups (Figure 12). FISS enlarged their volume 36-, 48-, 24- and 20-times within 14 days of treatment with NaCl, Au-GSH, Au-GSH-Dox, and Dox, respectively. In agreement with those results, there were no statistically important differences in Ki-67 (*p* = 0.17) between the treatment groups. Ki-67 LI varies from 0–56% (mean of 15.0 ± 23.4 and 17.6 ± 21.6 for Au-GSH-Dox and Dox, respectively) (Figure 13 and Figure 14).

## 3. Discussion

The sizes of the Au-GSH and Au-GSH-Dox particles determined using DLS and TEM method were different. This was the effect of surface modification of the gold nanoparticles by doxorubicin and glutathione. However, the main fraction was ca. 5.5 and 12 nm for Au-GSH and Au-GSH-Dox, respectively. The peak related to the surface plasmon resonance was fuzzy. The maximum was located at ca. 520 nm (Figure 2). This clearly confirms that metallic gold was formed [14,15]. Moreover, the fuzzy surface plasmon resonance peak located at 520 nm (Figure 2) also confirms that the core of gold nanoparticles is ca. 4 nm in diameter [16]. The zeta potential of the particles tested were equal to −52.9 ± 10.6 mV and −24.6 ± 11.9 mV for Au-GSH and Au-GSH-Dox, respectively. The thicker the electric double layer is, the higher the observed zeta potential absolute value can be [17]. The strong negative zeta potential was probably related to the doxorubicin present at the surface of Au-GSH. The zeta potential indicates negative surface charge properties of the nanoparticles [18]. It should be pointed out that the strong surface charge confirms that the colloid is stable and can be stored for a long time. Despite high stability, strong negative zeta potential suggests low toxicity of the nanoparticles to normal cells [19].

FISS growth from the FFS1 cell line in a murine model were grade III with very fast growth and tumor enlargement (Figure 12), which is in agreement with the previous studies on the chick embryo chorioallantoic membrane model [20]. Some of the tumors were attached to the vertebrae or scapula of the mice, indicating the highly invasive nature of FISS, which is in agreement with the clinical observations in cats [1,21]. Most of the tumors (35/40), both in the control and test groups, had an area of necrosis at the tumor core (Table 1), which indicates that necrosis was not a result of either Au-NP or Dox injections but appeared as a typical histopathological hallmark of FISS [13,21,22]. Recently, it has been demonstrated that necrosis is a sign of tumor malignancy, which fuels drug resistance and tumor progression [3,23]. Blood vessels were localized, mostly peripherally (Figure 3A), leading to inefficient oxygen supply at the tumor center. A specific tumor microenvironment appears due to heterogeneity in the blood supply, which negatively influences drug delivery, resulting in an inferior response to treatment [3].

After a single intravenous (IV) injection, Au-GSH demonstrated widespread organ distribution with the main accumulation in the liver and spleen (Figure 6), which is in agreement with other studies on organ distribution for Au-NPs smaller than 15 nm in diameter and is probably due to the uptake by the resident phagocytes [24,25,26,27,28]. The strong negative zeta potential of Au-GSH (Figure 2), essential for high colloid stability, may be a reason for the fast uptake of the gold nanoparticles by the mononuclear phagocyte system after IV administration [6]. According to the study performed by Keene and collaborators, if less than 2% of Au-NPs accumulate in the liver, it is an exclusion criterion in mouse models because of a “missed dose”. In the present study, at least 20% of Au-GSH was found in the liver of each mouse, which confirms proper IV injection of test compounds. However, extremely low accumulation within the tumor tissue after IV injection (less than 0.2% id/mg), together with positive results of previously performed in ovo studies [29], validates the intratumoral administration of Au-GSH. Various advantages such as fewer negative-side effects and a greater accumulation of the drug in cancer tissue following intratumoral delivery, in comparison to systemic routes, have been reported for various drug-loaded nanoparticles [30,31,32]. Gold nanoparticles were shown to have a size-dependent rat skin permeation in vitro, indicating 15 nm gold nanoparticles as a promising DDS for intradermal drug delivery [18]. Furthermore, in the study the presented intratumoral Au-GSH injections did not cause necrosis at the place of injection (as opposed to free Dox) and had no side effects on the liver and kidney parameters in the nude mice, which enables the intratumoral use of Au-GSH as a DDS for anticancer agents. Similarly, the lack of toxic effects of Au-NPs on blood biochemistry in mice was previously reported [33].

Understanding both the biological and physical properties of drug transport within solid tumors, recently termed “transport oncophysics”, is a clue for the development of efficient anticancer agents [34]. Deposits of Au-GSH were visible at the periphery as well as within the necrotic area within the center of the tumors. This observation suggests that Au-GSH may act as a DDS for standard chemotherapeutics that are unable to penetrate the tumor core due to specific physical properties of solid tumors such as high interstitial pressure, high cell density, or high collagen content in the tumor extracellular matrix [4,35,36]. The Au-GSH distribution within FISS is in agreement with most of the studies presenting Au-NP accumulation in the well-vascularized tumor regions, as well as their ability to rapidly penetrate the tumor core via diffusion in the tumor interstitial matrix or transport by macrophages [35,37]. Au-NP precipitates were always visible close to Dox in Au-GSH-Dox-treated mice, followed by a significantly (*p* ≤ 0.01) higher number of TUNEL-positive cells detected in the tumor core, in comparison to the periphery of the tumors, indicating its higher distribution at that area (Figure 10 and Figure 11).

Despite improved transport of Au-GSH-Dox to the tumor core, even with Au-NPs as the DDS, Dox did not reach all cancerous cells, which could be one of the causes of its failure as a treatment in mice as opposed to previously reported in vitro studies on FISS [12]. In vitro drug efficacy of Au-GSH-Dox in FISS, where all neoplastic cells are exposed to test compounds, may not correspond to its in vivo efficacy where tumor biology and the unique tumor microenvironment play an important role in drug transport and tumor progression. As opposed to the results obtained, Zhang and collaborators demonstrated greater efficacy of intratumorally injected Dox conjugated to Au-NPs than free Dox for a human melanoma xenograft in a murine model. On the other hand, therapeutic failure may be also closely related to the biological behavior of FISS and is hypothesized to be two-fold. Firstly, the large area of necrosis, which is a biological specification of high-grade FISS, results in the lack of Dox efficacy in low proliferating cells. Secondly, the high immunoexpression of TAM localized to this area promotes tumor progression and enhances drug resistance, as Dox is effective in highly proliferative cells due to its binding of topoisomerase II and blocking of DNA. TAM is generally known to more closely resemble M2-polarized macrophages activated by Th2 cytokines [38], secrete VEGF and antibiotic peptide PT38, and up-regulate the expression of various transcription factors (e.g., HIF1 and HIF2). This leads to tumor progression due to an increase in angiogenesis and to promoting tumor cells to adapt and survive hypoxia and increase resistance to standard chemotherapeutic agents [37,39,40,41,42]. Interactions between TAM and cancer stem cells lead to changes in tumor cell differentiation including the development of epithelial mesenchymal transition and cancer stem-like phenotypes [43]. The results of the studies on various human tumors (breast cancer, bladder cancer) indicate that TAMs are now being recognized as potential therapeutic targets.

In veterinary medicine, high immunoexpression of TAM (MAC 387) was shown to positively correlate with high metastatic ability in canine mammary tumors, as well as with poor prognosis [44,45,46]. Until now, to the best knowledge of the authors, there are no studies on the role of TAM in tumor progression and chemotherapy resistance in cats. The large area of necrosis and high immunoexpression of TAM, indicating “pro-tumor macrophages”, may be responsible for FISS tumor progression and therapeutic failure, making TAM a potential target for the effective treatment of FISS. However, further studies, both on the molecular level and their correlation with therapeutic outcomes in the clinic (e.g., with pexidartinib treatment), are required to test this hypothesis especially since it is impossible to extrapolate observations from one tumor type to another due to variations in the biological behavior [47]. What is of primary importance is that Au-GSH was shown to co-internalize with TAM close to the area of necrosis and in the tumor periphery. Therefore, the role of Au-NPs as a specific tool in targeting TAM and thus enhancing the response to conventional chemotherapy should be further investigated.

## 4. Materials and Methods

### 4.1. Cell Cultivation

The feline fibrosarcoma cell line with high P-gp activity (FFS1) used in this study was derived by Erichsen and co-workers [48]. Cells were grown in standard conditions (37 °C, 5% CO_2_) in Dulbecco’s Modified Eagle Medium (DMEM) supplemented with (*v*/*v*) heat-inactivated 10% fetal bovine serum (FBS), penicillin and streptomycin (50 IU/mL, Sigma Aldrich, Poznań, Poland), amphotericin B (2.5 mg/mL, Sigma Aldrich), and Plasmocin prophylactic (5 µg/mL, InVivoGen, San Diego, CA, USA). When the confluence reached 70–80%, cells were trypsinized, washed twice in phosphate-buffered saline (PBS), counted with Countess II Automated Cell Counter (ThermoFisher Scientific, Darmstadt, Germany), resuspended in PBS, and kept on ice until administration into mice.

### 4.2. Au-GSH and Dox Conjugated to Glutathione-Stabilized Gold Nanoparticles (Au-GSH-Dox) Preparation and Characterization

Au-GSH (4.3 ± 1.1 nm) and Au-GSH-Dox were prepared according to the previously described procedure [12]. Briefly, gold chloride solution was added to a round-bottom flask and diluted with 26 mL of distilled water. The solution was stirred using a magnetic stirrer in an ice-cold bath for 30 min. Then, 162 mg of glutathione was slowly dispensed over a two-minute time period. The solution changed color from yellow through dark brown to white resulting in the formation of the gold polymeric intermediate. After 2 mL of saturated sodium bicarbonate (NaHCO_3_) was added, the solution turned clear. After 15 min, a freshly made aqueous solution (50 mg in 6.5 mL of distilled water) of sodium borohydride (NaBH_4_) was added under vigorous stirring and the reaction changed color into dark brown, the result of nanoparticle formation. The reaction was left to proceed for two hours. To purify the solution, 32 mL of methanol (MeOH) was added under stirring conditions. The precipitants were collected by centrifugation (6000 RPM, 10 min), washed with 1 mL of MeOH/H_2_O (1:1, *v*/*v*), and three times with methanol (1 mL × 3) and dried under reduced pressure at room temperature.

**Figure 15:**
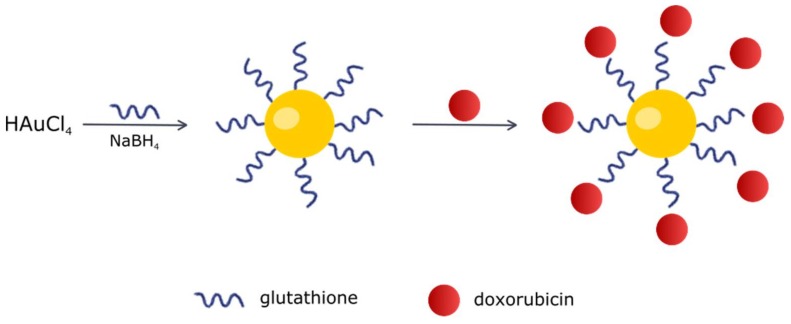
Scheme of Au-GSH-Dox preparation.

To prepare Au-GSH-Dox (Figure 15), 20 mg of solid Au-GSH was dissolved in 2 mL of distilled water. Then, 1 mL of Dox hydrochloride solution (1 mg/mL) was slowly added while mixing. The solution was left for 1 day in order to obtain the non-covalent conjugate. Then, purification was performed by centrifugation using a centrifuge filter. The material was rinsed with phosphate buffer until there was no free Dox in the filtrate. The Au-GSH: Dox ratio in Au-GSH-Dox was 20:1, which was previously confirmed by small angle X-ray scattering (SAXS) analyses [12].

The solutions obtained after each step of synthesis (i.e., Au-GSH, Au-GSH-Dox) were collected and analyzed spectrophotometrically using Shimadzu model U-2501PC. The hydrodynamic radii and zeta potentials were determined using the Malvern Zetasizer Nano ZS. For this purpose, a standard clear polycarbonate cell with gold electrodes was applied.

### 4.3. Animal Studies

All procedures described above were performed in agreement with the approval of the II Local Animal Ethic Commission of the Warsaw University of Life Sciences (decisions No. 39/2014 (25 September 2014), 83/2015 (17 December 2015) and WAW2/55/2017 (21 June 2017)). Studies were performed according to the “3R” guidelines with respect to animal welfare. Human endpoints were implemented and included tumor necrosis, tumor size above 1500 mm^3^, acute weight loss, and behavioral changes indicating pain or severe distress.

#### 4.3.1. Animal Housing

In total, forty-six 5-week-old male immunodeficient BALB/cAnNRjFoxn1nu/Foxn1nu mice (Envivo, Vivari, Warsaw, Poland) were utilized in these studies [49]. After arriving at the facility, the animals were kept in groups of four or five in individually ventilated cages (GMC 500 Mouse IVC Green Line, Tecniplast, Buguggiate, Italy) (temperature 22 °C, relative humidity 55 ± 10%, with air changes 75 times per hour), fed with mouse breeding high-energy sterilized 25 kGy diet (ssniff, ssniff Spezialdiaten GmbH, Soest, Germany), and given water ad libitum. A diurnal rhythm was maintained with a 12:12 h light–dark cycle with artificial light from 8:00 a.m. After seven days of acclimatization, mice were divided into single cages for experimentation. All procedures were performed under a laminar flow hood (BS 60 biosafety changing station, Tecniplast, Italy).

#### 4.3.2. Au-GSH and Au-GSH-Dox Distribution and Efficacy Studies

Feline fibrosarcoma cells (3 × 10^6^ FFS1 cells in 20 uL of PBS) were subcutaneously injected using 100 IU Micro-Fine Plus insulin syringe (0.8 mm × 30 G) (Beckton Dickinson, Warsaw, Poland) in the intrascapular region of the mice. The intrascapular region, not inguinal, was selected for injection as it is a typical area of localization for FISS.

When tumors reached 100 mm^3^, six mice were injected intravenously (IV) via the tail vein with Au-GSH (40 mg/kg). Twenty-four hours later mice were euthanized via cervical dislocation and organs (tumors, liver, spleen, kidney, heart) were collected. Organs were washed twice with 0.9% sodium chloride (NaCl) and placed at −80 °C before running Au-GSH and Au-GSH-Dox distribution analyses with an inductively-coupled plasma mass spectrometer (ICP-MS).

The rest of the animals were divided into 4 groups and the following test compounds were injected intratumorally three times a week (every Monday, Wednesday and Friday): 0.9% sodium chloride (NaCl) (control group; *n* = 7), Au-GSH (50 mg/kg; *n* = 7), Dox (2.5 mg/kg; *n* = 13), and Au-GSH-Dox (Dox: 2.5 mg/kg, Au-GSH 50 mg/kg*; *n* = 13). On the same days, tumor volume was calculated using the following equation: V = (*w*)^2^ × (*l*)/2, where (*w*) and (*l*) are the width and length of the tumor as measured with an automatic caliper. Animal body weight was measured simultaneously. Animals were euthanized by cervical dislocation when any human endpoint was reached. Then, tumors and organs were harvested from animals, washed with PBS, weighed, and collected for histopathology, immunohistochemistry, and confocal microscopy. In addition, immediately after euthanasia, blood samples were collected from the vena cava into 0.5 mL tubes containing heparin and sent to the commercial reference Idexx laboratory for assessment of creatinine and blood urea nitrogen (BUN) to assess kidney function, and alanine transaminase (ALT) and asparagine transaminase (AST) to assess liver damage.

### 4.4. ICP-MS Analyses

Au-GSH distribution in organs was measured via ICP-MS analyses, according to a previously published protocol [25,50]. Briefly, gold extraction from selected organs involves burning of the tissues and then dissolving the residue in aqua regia. The burning process was performed in several steps. In the first step, samples were heated to 200 °C within 3 h, which makes it possible to slowly remove water. Next, samples were heated to 700 °C. The heating process was carried out over 3 h. Then, samples were kept at this temperature for next 6 h. This final step effectively incinerates the samples. Subsequently, samples were cooled to room temperature. The obtained ash was dissolved in aqua regia using 3 mL per sample in order to dissolve the metallic gold. The mixture of a concentrated nitric acid (65%, Avantor, p.a.) and hydrochloric acid (37%, Avantor, p.a.) in a volume ration 1:3 was utilized to prepare aqua regia. The metal concentration was determined using a spectrometer, the 4200 MP-AES Agilent (Microwave induced Plasma-Atomic Emission Spectrophotometer, Agilent, Santa Clara, CA, USA). Each sample was analyzed in triplicate.

### 4.5. Confocal Microscopy

Tissue sections were fixed in 3% paraformaldehyde, dehydrated by passages through ethanol solutions (70%, 80%, 90%, 96%, 100% (two times) for 10 min each), cleared in absolute xylene (two times for 10 min each), mounted in DPX (Gurr, Sigma Aldrich, Poznań, Poland), and covered with 1.5 coverslips. ZEISS AxioObserver.Z1 microscope (Carl Zeiss, Oberkochen, Germany) with LSM 780 confocal system was used to assess the localization of Dox in Au-GSH-Dox and Dox-treated tumors within the sections. Images were collected through the 3-channel procedure (bright field in transmitted channel (TPMT detector) for Au-GSH visualization; green fluorescence: excitation 488 nm and detection over 540 nm for visible tissue auto-fluorescence; and red fluorescence: excitation with 561 nm laser and detection over 623 nm for Dox auto-fluorescence) in combination with objective EC Plan Neofluar 10×/0.3. Two different regions of each tumor (peripheral and the core) were chosen for image analysis performed using a ZEN 2.3 microscope and imaging software (blue edition, Carl Zeiss, Oberkochen, Germany). Tumors treated with NaCl and Au-GSH were used as a negative control for optimization of laser intensity and detector gain settings, which were applied for all analyzed samples.

### 4.6. Histopathology and Immunohistochemistry

The tissue samples were fixed in 10% formalin for approximately 48 h, embedded in paraffin blocks, then cut into 3 μm sections, and routinely stained with hematoxylin and eosin (HE) to confirm the hallmarks of FISS. Tumor grade was assessed based on mitotic rate (determined as the mean number of cells in mitosis evaluated in 10 high-power fields (HPF) under 40× objective lens), cellular differentiation, and the presence and extension of necrosis within the tumor, according to a scale proposed by Couto and co-workers [13].

All tumors were examined for tumor-associated macrophages (TAMs), according to the procedures published by Król and collaborators (MAC 387, dilution 1:1000, Dako, Glostrup, Denmark) [45], to assess their role in tumor progression and the distribution of gold nanoparticles.

To indirectly assess tissue localization of the drug by detecting the effect of the drug on cells the terminal deoxynucleotidyl transferase–mediated dUTP nick end labeling (TUNEL) assay with Apo-Brdu-IHC in situ DNA fragmentation kit (Biovision, Mipitas, CA, USA) following the uptake of BrdU into cells during the S phase as an apoptosis marker, was performed according to the manufacturer’s protocol. The number of apoptotic cells per HPF was calculated manually in the regions of interest (ROI): tumor core and tumor periphery. At least 10 HPF for each region were counted. Both the necrotic area and inflammatory cells were omitted. The numbers of apoptotic cells are presented as the mean with standard error of the mean (SEM).

Immunohistochemistry for Ki-67 (clone MIB-1, dilution 1:75, Dako, Denmark) was performed as previously described [20] to confirm the lack of the influence of tested compounds on proliferation markers. Samples were analyzed in a quantitative way using light microscopy (Axio Imager A2, Carl Zeiss, Oberkochen, Germany). For each individual analysis, paraffin-embedded tissues of feline lymph nodes were used as the positive control, while staining without the use of primary antibody was used as a negative control. Ki-67 LI were calculated manually as the number of positively stained tumor cells among the total number of tumor cells. Both the necrotic area and inflammatory cells were omitted. Ki-67 LI are presented as the mean with standard deviation (SD).

### 4.7. Statistical Analyses

Statistical analyses of Au-NP concentration in organs, immunoexpression of Ki-67, and blood parameters (creatinine, BUN, AST, ALT) among the treated groups were performed with one-way analysis of variance (ANOVA) and post-hoc Tukey test (GraphPad Prism version 5.0, GraphPad Software, La Jolla, CA, USA). Differences in TUNEL-positive cells between ROI were assessed with the Student’s *t*-test (GraphPad Prism version 5.0, GraphPad Software, La Jolla, CA, USA). Each data is presented as mean ± SD or mean ± SEM. *p* ≤ 0.05 (*) was assigned as significant, while *p* ≤ 0.01 (**) or *p* ≤ 0.001 (***) were assigned as highly significant.

## Figures and Tables

**Table 1:** Histopathological characterization of feline injection site sarcomas (FISS) growth in a murine model from FFS1 cell line.

Tested Compound	ID	Grading System of Fibrosarcoma ^1^					Hemorrhages	Inflammation	Au-GSH Deposits
		Differentiation	Mitotic Count	Mitotic Score	Necrosis	Grade			
NaCl	93795	3	148	3	1	III	Yes	1	No
NaCl	93864	3	103	3	3	III	Yes	0	No
NaCl	93869	3	133	3	3	III	Yes	1	No
NaCl	93870	3	90	3	3	III	Yes	3	No
NaCl	93872	3	124	3	2	III	Yes	0	No
NaCl	93873	3	144	3	3	III	Yes	1	No
NaCl	93874	3	139	3	3	III	Yes	0	No
Au-GSH	93862	3	113	3	1	III	Yes	0	Peripherally
Au-GSH	93863	3	164	3	3	III	Yes	1	Peripherally and in tumor within area of necrosis
Au-GSH	93866	3	121	3	2	III	Yes	0	Peripherally
Au-GSH	93867	3	121	3	2	III	Yes	1	Peripherally and in tumor within area of necrosis
Au-GSH	93871	3	191	3	3	III	Yes	1	Peripherally and in tumor within area of necrosis
Au-GSH	93793	3	121	3	2	III	Yes	0	Peripherally
Au-GSH	93794	3	116	3	2	III	Yes	3	Peripherally and in tumor within area of necrosis
Au-GSH-Dox	93842	3	111	3	3	III	Yes	2	Peripherally
Au-GSH-Dox	93843	3	99	3	3	III	Yes	3	Peripherally and in tumor within area of necrosis
Au-GSH-Dox	93844	3	59	3	2	III	Yes	1	Peripherally and in tumor within area of necrosis
Au-GSH-Dox	93868	3	124	3	3	III	Yes	1	Peripherally
Au-GSH-Dox	93846	3	101	3	2	III	Yes	0	Peripherally and in tumor within area of necrosis
Au-GSH-Dox	93847	3	142	3	2	III	Yes	0	Peripherally and in tumor within area of necrosis
Au-GSH-Dox	93848	3	136	3	2	III	Yes	2	Peripherally and in tumor wthin area of necrosis
Au-GSH-Dox	93849	3	84	3	3	III	Yes	3	Peripherally
Au-GSH-Dox	93851	3	111	3	3	III	Yes	1	Peripherally and in tumor within area of necrosis
Au-GSH-Dox	93858	3	115	3	3	III	Yes	0	Peripherally and in tumor within area of necrosis
Au-GSH-Dox	93853	3	100	3	2	III	Yes	3	Peripherally and in tumor within area of necrosis
Au-GSH-Dox	93788	3	54	3	2	III	Yes	1	Peripherally and in tumor within area of necrosis
Au-GSH-Dox	93789	3	98	3	2	III	Yes	2	Peripherally
Dox	93791	3	114	3	2	III	Yes	0	No
Dox	93854	3	108	3	2	III	Yes	0	No
Dox	93855	3	125	3	2	III	Yes	2	No
Dox	93856	3	134	3	3	III	Yes	3	No
Dox	93857	3	101	3	2	III	Yes	2	No
Dox	93858	3	92	3	1	III	Yes	0	No
Dox	93859	3	63	3	1	III	Yes	1	No
Dox	93860	3	96	3	2	III	Yes	1	No
Dox	93861	3	112	3	2	III	Yes	1	No
Dox	93845	3	108	3	2	III	Yes	1	No
Dox	93850	3	101	3	2	III	Yes	1	No
Dox	93865	3	155	3	1	III	Yes	1	No
Dox	93792	3	169	3	2	III	Yes	1	No

^1^ According to Couto et al. grading system, based on mitotic rate, cellular differentiation and the presence and extent of necrosis within the tumor [13].

## Abbreviations

Au-NP	gold nanoparticle
Au-GSH	glutathione-stabilized gold nanoparticle
Au-GSH-Dox	doxorubicin conjugated to glutathione-stabilized gold nanoparticles
ALT	alanine transaminase
AST	asparagine transaminase
BUN	blood urea nitrogen
DDS	drug delivery system
DLS	dynamic light scattering
Dox	doxorubicin
FISS	feline injection-site sarcoma
HE	hematoxylin and eosin
HP	histopathology
HPF	high power field
ICP-MS	inductively-coupled plasma mass spectrometer
IV	intravenous
MDR	multidrug resistance
MeOH	methanol
MRP	multidrug resistance protein
NaBH_4_	sodium borohydride
NaCl	Sodium chloride
NaHCO_3_	saturated sodium bicarbonate
PBS	phosphate-buffered saline
P-gp	P-glycoprotein
SAXS	small angle X-ray scattering
SD	standard deviation
SEM	standard error of mean
TAM	tumor-associated macrophages
